# Gender Differences in the Incidence and Types of Sports Injuries Among Female Athletes: A Scoping Review

**DOI:** 10.1155/jonm/6416101

**Published:** 2025-08-30

**Authors:** Liang Sun, Lanfang Luo, Yi Yang, Qiushui Wangchuan, Jiong Luo

**Affiliations:** ^1^College of Physical Education, Southwest University, Chongqing, China; ^2^Department of Physical Education, Chongqing Mining Engineering School, Chongqing, China

**Keywords:** female athletes, gender differences, nursing strategy, scope review, sports injuries

## Abstract

**Background:** With the increasing participation of women in sports, understanding gender differences in sports injuries has become crucial. This study compares the incidence of sports injuries in male and female athletes through a scope review, analyzes the gender specific patterns of common injury types, summarizes the current situation and challenges of sports injury prevention in female athletes, and proposes targeted clinical practice and research recommendations.

**Method:** This study adopted a scope review design, followed the PRISMA ScR guidelines, and registered with PROSPERO (CRD420251058146). By searching PubMed, CINAHL, Embase, Web of Science, and Cochrane databases through the system (as of May 23, 2025), and manually searching for relevant literature in conjunction with references. The inclusion criteria are based on the PICO framework, focusing on female athletes, gender factors, and the incidence of sports injuries, covering all types of research. The data were extracted into standardized tables by two independent researchers through two-stage screening (title/abstract and full text).

**Result:** Eighteen studies were selected from 2487 articles, covering various sports and populations. The results showed that there was no significant difference in the overall incidence of sports injuries between male and female athletes. However, female athletes are at higher risk in specific types of injuries, such as ACL injuries, bone stress injuries, concussion, and nontraumatic shoulder instability. The study also found that women are underrepresented in sports science and medical research, and gender factors are often overlooked.

**Conclusion:** Although the overall incidence of injuries is similar, female athletes face higher risks in specific types of injuries and require targeted prevention strategies. Key research challenges include the underrepresentation of women and the neglect of social gender factors. It is recommended to integrate both sex-based biological factors and gender-related social factors into sports injury prevention models to optimize practice. For nursing management, these findings highlight a pivotal role in mitigating injury risks. Nurse managers should champion educational programs on female-specific risks, lead the implementation of screening protocols, foster interdisciplinary collaboration, and advocate for research that addresses the gender gap in sports medicine.

## 1. Introduction

With the increasing number of women participating in sports globally, issues related to female sports injuries have received more attention than before [1]. The increase in sports participation may lead to an increase in the incidence of sports-related injuries. For example, Caroline et al. [2] found that the number of cases of hospitalization due to sports injuries increased by 24% in 7 years (from 2004 to 2010), with an annual growth rate of approximately 3.1%. Therefore, it is of great significance to pay attention to issues related to female sports injuries. The attention paid to issues in female sports science and sports medicine is relatively insufficient compared to that of men [3, 4]. Previous literature has collated relevant data from six authoritative journals in the field of sports science in the past seven years (from 2014 to 2020) and found that among all 5271 published publications, less than 40% of the subjects were female, while more than 60% were male [5]. To a certain extent, this reflects that most research conclusions may only be applicable to men. Therefore, attention to related issues of women should be increased.

To discuss the factors contributing to sports injuries, it is crucial to distinguish between an athlete's biological sex and their socially constructed gender. Sex-based factors are physiological and anatomical, including hormones, genetics, and body structure. In contrast, gender-related factors are shaped by social roles, cultural norms, and societal expectations. [6]. These two aspects of differences may lead to different injury occurrences among male and female athletes. Currently, there is no unanimous conclusion on this issue [7–9]. Although a large number of previous studies have discussed sports injury risk factors in detail, most of them remain at the individual physiological level, and the influence at the social level has been less concerned and discussed [10, 11].

Currently, there remains a notable lack of consensus and comprehensive evidence regarding gender-based comparisons of sports injury epidemiology and detailed analyses of sex-specific differences in common injury patterns. This study systematically synthesizes the current state of knowledge and identifies key challenges in injury prevention practices and research specific to female athletes. The findings establish a crucial foundation for developing gender-tailored injury prevention strategies and formulating targeted research priorities to address existing gaps in sports medicine.

## 2. Aim

Through this scoping review, we aim to conduct a meticulous comparison of sports injury incidence between male and female athletes, thoroughly analyze gender differences in common sports injury types, comprehensively summarize the current status and challenges of sports injury prevention practices and research pertaining to female athletes, and propose targeted clinical practice and research recommendations based on existing evidence. These investigations are expected to elucidate the relationship between gender factors and sports injury occurrences, while facilitating a gender-informed reevaluation of sports injury prevention strategies.

## 3. Methodology

### 3.1. Design

A scoping review represents a form of knowledge synthesis that systematically maps the existing literature by searching, selecting, and synthesizing available evidence. It serves to outline key concepts within a field, identify research trends, characterize the diversity of existing knowledge, and inform future research directions [12]. This study strictly adhered to the Preferred Reporting Items for Systematic Reviews and Meta-Analyses extension for Scoping Reviews (PRISMA-ScR) guidelines as outlined by Tricco et al. [13]. The systematic review protocol was registered in the International Prospective Register of Systematic Reviews (PROSPERO) under registration number CRD420251058146.

### 3.2. Search Methods

This study rigorously followed the updated methodological framework for scoping reviews proposed by Peters et al. [14], which aligns with the PRISMA-ScR guidelines [13] to ensure reporting consistency. Various concepts, measurement tools, and well-being indicators related to caregivers were systematically mapped through distinct phases (identifying research questions, determining relevant studies, study selection, data charting, collation, summarization, and result reporting). This process helped elucidate key factors and interventions supporting research in health leadership.

Based on a comprehensive review of existing literature, we formulated four research questions:

• What are the differences in sports injury incidence between male and female athletes?
• What gender-based variations exist in common types of sports injuries?
• What challenges and limitations currently exist in sports injury prevention practices and research for female athletes?
• What targeted clinical and research recommendations can be proposed to address these gaps?

### 3.3. Identifying Relevant Studies

We systematically evaluated relevant studies retrieved from the following computerized databases: PubMed, CINAHL, Embase, Web of Science, and Cochrane. Additionally, we manually reviewed the reference lists of all included studies to identify potentially eligible publications that may have been missed in the initial search. The search period encompassed records from database inception through May 23, 2025. The search used terms related to gender, sports injury epidemiology, and female athletes, such as “sex or gender,” “sports injury epidemiology,” and “female athletes.” A complete list of search strategies and terms can be found in Supporting Document 1.

### 3.4. Inclusion and Exclusion Criteria

The inclusion and exclusion criteria were established according to the PICO framework [14], where P represents the population (female athletes), I denotes the intervention (gender factors), C indicates the comparison (male athletes), and O refers to the outcomes (sports injury incidence, gender-specific differences, and injury prevention strategies). This study uses the PICO framework to answer the following questions: What are the gender-specific sports injury risk factors in female athletes? This framework guided literature search and analysis to ensure that the studies were systematic and targeted.

Inclusion criteria:

• Studies focusing on female athletes.
• Research primarily addressing sports injury incidence, gender-specific variations, and injury prevention strategies in female athletes.
• Publications in the English language.
• All study designs.

Exclusion criteria:

• Duplicate publications.
• Studies with unavailable full-text access.
• Conference abstracts without full manuscripts.

### 3.5. Charting the Data

All identified literature records were imported into EndNote X9 (Clarivate Analytics) for duplicate removal. Two independent investigators performed a two-stage screening process according to the predefined eligibility criteria. The initial screening stage involved title and abstract review, followed by full-text assessment of potentially eligible articles in the secondary stage. Any discrepancies between reviewers regarding study inclusion were resolved through discussion with a third senior researcher until consensus was achieved. Following the final selection, we extracted relevant data from included studies and populated a standardized data extraction form.

## 4. Results

### 4.1. Bibliographic Overview

**Figure 1:**
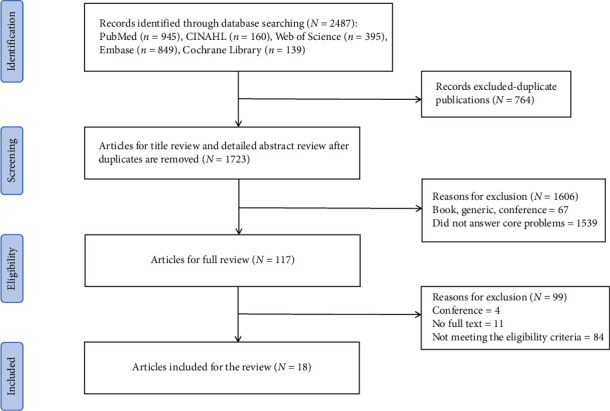
PRISMA flow diagram of the article selection process.

Our systematic search of electronic databases yielded 2487 potentially relevant articles. Following the removal of duplicates, we screened 1723 records based on title and abstract. Subsequently, 117 full-text articles were assessed for eligibility, of which 99 were excluded for various reasons. Ultimately, 18 studies met our inclusion criteria and were incorporated into this scoping review (see Figure 1). The baseline characteristics of the included studies are presented in Table 1.

### 4.2. Current Status of Female Sports Injury Prevention

#### 4.2.1. Types of Sports Injuries and Gender Differences

There are primarily two statistical methods for sports injury events [28]. One is the statistical method based on the occurrence of sports injuries, which includes incident cases (ICs), referring to the number of new injury cases that occur within a certain period, usually a season; incidence rate (IR), representing the ratio of the number of new injuries to the athlete exposure time during a tracking period, such as the number of sports injuries per 1000 athlete-exposures (1000 A-E) or per 1000 athlete-hours (1000 A-H); and incidence proportion (IP), that is, the number of new cases of a particular injury divided by the total number of all athletes. The other is the statistical method based on the prevalence of injuries, including prevalent cases (PCs), referring to the total number of injury cases that occur at a specific time point or within a period; prevalence proportion (PP), referring to the ratio of the number of all injured athletes to the total number of all athletes within a period [29, 30]. Nielsen et al. [28] pointed out that when studying the etiology, prevention, or treatment of sports injuries, the statistical method based on the occurrence of sports injuries is recommended. Therefore, in this study, when collating the occurrence of sports injuries, only the injury occurrence cases (IC), injury IR, or injury occurrence proportion (IP) were used as the data for injury recording.

This article conducted a literature search in the electronic database Google Scholar using the keywords “sex or gender,” “sports injury epidemiology,” and “female athletes.” By integrating the research results and conclusions of relevant literature studies, it was found that the occurrence of sports injury events among male and female athletes varies in different athlete sample groups. For example, the overall injury occurrence of all male and female athletes in a school differs from that of male and female athletes engaged in individual sports.

Specifically, there is no significant difference in the overall occurrence of sports injuries between male and female athletes. Sallis et al. [8]counted the sports injury data of the American university sports department over the past 15 years and found that among 3767 male and female athletes engaged in seven sports including football, basketball, track and field, swimming, tennis, cross-country, and water polo, the average sports injury occurrence proportion in 12 different parts of the body was 52.45 times/(100 people/year) for females and 47.68 times/(100 people/year) for males, with no statistically significant difference. Frisch et al. [8] found that among high-level athletes under 19 years old engaged in 12 different sports, the sports injury IRs of boys and girls were 1.20 times/1000 A-H and 1.21 times/1000 A-H, respectively, with no significant difference. Comstock et al. [15] collated the sports injury data of American high school students from 2005 to 2006 and found that there was no significant difference in the sports injury IR between male high school students engaged in baseball, football, wrestling, basketball, and rugby and female high school students engaged in softball, volleyball, basketball, and football.

However, for athletes engaged in specific sports, there are significant differences in the occurrence of sports injuries between men and women. Sallis et al. [8] found that although there was no difference in the sports injury occurrence proportion of male and female college athletes overall, in swimming and water polo, the sports injury IRs of male and female athletes were significantly different: The sports injury occurrence proportion of female swimmers was 47.08 times/(100 people/year) and that of males was 12.37 times/(100 people/year), and the shoulder joints, backs, hip joints, and knee joints of female swimmers were more likely to be injured; the sports injury occurrence proportion of female water polo players was 18.38 times/(100 people/year) and that of males was 7.10 times/(100 people/year), and the shoulder joints of female water polo players were more likely to be injured. Frisch et al. [7] pointed out that in team competitive sports, the probability of girls suffering sports injuries is 2.05 times that of boys, and the probability of ankle injuries is greater. In particular, a 6-year cohort study pointed out that the sports injury IR of male athletes participating in the Alpine Skiing World Cup in training and competition was significantly higher than that of female athletes, which were 39.7 times/100 people/season and 31.9 times/100 people/season, respectively (relative risk [RR] = 1.24, 95% CI 1.05 to 1.47) [9].

Different sports patterns, movement characteristics, and injury mechanisms may be the potential reasons for the differences in the occurrence of injuries among male and female athletes in different sports, so it is necessary to conduct a targeted discussion rather than making generalizations. For sports with a high incidence of sports injuries and injury sites, the discussion should be based on the characteristics of the sport and combined with the anatomical, physiological, and biomechanical characteristics of the injured site. At the same time, gender and age differences should also be considered. For example, due to different physiological, psychological, and social changes caused by physical development, male and female athletes of different age groups have different impacts on the occurrence of sports injuries.

#### 4.2.2. Analysis of the Scarcity Dilemma in Female Sports Science and Sports Medicine Research

Costello et al. [14] analyzed the proportion of male and female subjects in sports science research. They reviewed 1382 studies published in three well-known sports science journals from 2011 to 2013 and found that among 6.076 million participants, female subjects accounted for only 39% of the total. Studies only on females accounted for 4%–13% of the total studies in different journals, while studies only on male subjects accounted for 18%–34% of the total number of studies. This result indicates that the participation proportion of women in sports science and sports medicine research is significantly lower than that of men. However, this situation has not been improved. Cowley et al. [5] investigated six well-known sports science journals from 2014 to 2020, including 5261 studies with 12.51 million subjects and found that male subjects accounted for 66% of the total number, female subjects accounted for 34%; studies with both male and female subjects accounted for 63%, studies only with male subjects accounted for 31%, and studies only with female subjects accounted for only 6%. This shows that in recent years, there is still a large research gap for women in sports science and sports medicine research.

The reasons behind this phenomenon may include female hormone changes. For example, estrogen and progesterone fluctuate with the physiological cycle, resulting in more complex experimental design, longer experimental time, and higher experimental cost [31]; in addition, the recruitment of female athletes is also more difficult because the number of people in the sports team is small and the number of people who meet the experimental conditions is insufficient [32].

#### 4.2.3. Reflection on the Neglected Status of Gender Factors

The United Nations clarifies that distinctions should be made between biological sex and social gender. Biological sex refers to physical and physiological characteristics like hormones, genes, and anatomy, whereas social gender encompasses the identities, roles, and structures formed by social construction. Although sex-based differences have been widely studied, gender-related social factors have been largely overlooked, exacerbating the research gap concerning female athletes [18, 33, 34].

Consequently, traditional classification methods for sports injury risk factors are often deficient because they lack considerations of social gender. In a literature review describing the goals of acute sports injury prevention measures, most injury prevention strategies require changes in individual behavior and rarely involve policies, rules or the environment where injuries occur [10]. Specifically, the previous classification method of sports injury risk factors emphasizes the use of a dualistic approach including internal and external factors for classification [35]. Internal factors refer to relevant factors inside the athlete, such as anatomical structure, hormones, genes, and neuromuscular characteristics; external factors refer to factors in the external situation, such as weather conditions and equipment materials [10]. It can be seen from the table that most of the sports injury risk factors focus on the characteristics of the athletes themselves and ignore the influence of social construction. For example, for a long time, there have been stereotypes about male and female sports participants [36, 37], believing that women are not active enough in sports and cannot obtain sufficient sports resources like men or believing that women are naturally weak and cannot engage in intense sports or are more likely to be injured [38, 39]. Gender-differentiated parenting methods make girls relatively less encouraged to engage in more adventurous and independent physical activities [40]; the gym environment and culture are not conducive to the active development of muscle strength in women [17, 41, 42]; society does not expect or accept women to have well-developed muscles [43]; coaches of different genders adopt different training methods [44]. Sports injury management requires the cooperation of athletes, parents, coaches, and even all members of society.

### 4.3. The Intrinsic Relationship Between the Physiological Characteristics of Female Athletes and Sports Injuries

#### 4.3.1. Fluctuations in the Menstrual Cycle and Hormonal Linkage Effects

The impact of menstrual cycle phases (MCPs) on the injuries of female athletes has always attracted attention [22, 45, 46]. The continuous hormonal fluctuations during MCP seem to affect the material structure and mechanical properties of muscles [47], tendons [48], bones [22], and ligaments [49, 50]. The normal MCP includes the follicular phase before ovulation and the subsequent luteal phase [51–53]. The fluctuations of reproductive hormones in MCP are associated with an increased risk of acute [49, 50, 54, 55] and overuse [47, 48, 56] injuries. Studies have shown that the injury IR of female football players in the late follicular phase (47%) is higher than that in the early follicular and luteal phases (32%). In addition, compared with the luteal phase, the risks of muscle and tendon injuries in the late follicular phase increase [47, 55], and the risk of anterior cruciate ligament (ACL) injury also increases [3, 49]. A recent systematic review pointed out that the peak value of estradiol during ovulation is related to relaxation, poor strength, and neuromuscular control, which makes athletes more vulnerable to injury. However, the data are inconsistent and the direct causal relationship of the injury has not been determined.

The characteristics of menstrual irregularities include oligomenorrhea, menorrhagia, amenorrhea, anovulation, or lack of luteal phase [22, 46, 57], which are common among athletes in different sports and are associated with an increased risk of injury [22, 57]. In terms of time loss, high school athletes with menstrual irregularities suffer more severe injuries than those with normal menstruation. The female athlete triad (FAT) describes the interrelationship between menstrual irregularities, low energy utilization, and reduced bone density [6, 57]. Recently, FAT has been called relative energy deficiency in sport (RED-S), which is a condition of insufficient energy intake relative to energy consumption, resulting in impaired physiological functions, including but not limited to metabolic rate, menstrual function, bone health, immunity, and cardiovascular health. This is crucial for both male and female athletes, with particular emphasis on the significant health and performance impacts on female athletes [58, 59]. Some studies have investigated the relationship between menstrual irregularities and bone injuries and found that adolescents [1, 30] and adult athletes with current or previous menstrual dysfunction are particularly prone to bone stress injuries (BSIs).

#### 4.3.2. Interaction Between Human Anatomical Structure and Biomechanics

Women typically have a wider pelvis, larger knee abduction angles, greater knee abduction moments, higher vertical ground reaction forces, and smaller knee flexion angles compared to men. These anatomical and biomechanical differences alter lower limb alignment, increasing the risk of knee injuries.

Bone mineral density (BMD) is also affected by gender and increases the risk of stress fractures in female athletes. A study on 72 height- and weight-matched recruits [23] found that although the body sizes were comparable, male recruits had greater BMD in the hip and distal tibia than female recruits, which was related to thicker cortices. Another study [60] found that women have earlier and faster bone loss than men. Therefore, the prevalence of osteoporosis and osteoporotic fractures is significantly higher in women than in men. At the age of 50, the risk of osteoporotic fractures in women is 39.7%, while that in men is 13.1% [61]. Based on the gender differences in bone mass and geometry, men have a lower incidence of stress fractures and osteoporotic fractures [60]. Patellar instability is more common in women and is related to high patella, trochlear dysplasia, generalized ligament laxity, an increased angle between the tibial tuberosity and the center of the patella (Q-angle), and an increased distance from the tibial tubercle to the trochlear groove [62]. Female athletes are twice as likely to have ankle sprains as male athletes and have a higher probability of chronic ankle instability [63]. Among college athletes, the IR of stress fractures in women is about twice that in men. Compared with men, women have less muscle mass, are shorter, and have lower BMD [62], all of which also increase their risk of sports injuries. Through the above analysis, it can be seen that the gender differences in physiological structure and biomechanical characteristics significantly affect the sports injury risk of female athletes, which provides a scientific basis for formulating targeted prevention and treatment strategies.

#### 4.3.3. Catalytic Effect of Psychological Stress, Anxiety, and Depression on Sports Injuries

Recently, a cross-sectional observational study on adult athletes in the elite system [24] showed that female athletes have a higher incidence of mental health symptoms and a higher proportion of poor mental health. Compared with male athletes, female athletes have a higher proportion of self-reported interpersonal conflicts, economic difficulties, and negative discrimination. Female athletes are twice as likely to have depressive symptoms as male athletes [64] and also have higher incidences of anxiety [65] and eating disorders [64]. Generally speaking, the estimated prevalence of eating disorders and abnormal eating symptoms in the athlete group is 6%–45% in women [66], which is much higher than that in men (0%–19%) [67]. As many as 60% of elite female athletes said that they have suffered from body shaming pressure from coaches [68]. Due to concerns about stigmatization, embarrassment, poor mental health knowledge, and privacy protection, both male and female athletes have a more negative attitude toward seeking help than nonathletes [69].

The mechanism by which psychological stress, anxiety, and depression affect sports injuries is a complex and crucial topic. Particularly among female athletes, the relationship between psychological factors and sports performance as well as injury risk is especially prominent. Research indicates that psychological stress not only impacts an athlete's performance but may also directly lead to sports injuries [70]. Psychological stress triggers a series of physiological responses. Under stress, muscles contract involuntarily, resulting in muscle fatigue and reduced coordination, thereby increasing the risk of injury. Stress also affects the function of the nervous system, leading to decreased attention and reaction speed and enhancing the likelihood of sports injuries. Additionally, psychological stress causes an increase in stress hormones such as cortisol. Long-term high levels of cortisol are associated with immunosuppression, meaning that athletes are more prone to infection and injury under high pressure [71]. Hormonal fluctuations may lead to emotional instability, which exacerbates psychological stress and forms a vicious cycle. In a high-pressure environment, an athlete's decision-making ability may be affected. A strong stress reaction can reduce an athlete's judgment and decision-making capabilities, making it impossible for them to make the optimal choice during a competition, resulting in technical errors and an increased risk of injury. The psychological state after injury is also significant. Injured athletes typically experience emotions such as anxiety, depression, and self-doubt, which may influence their rehabilitation process. Excessive psychological stress may prolong the recovery time and even lead to a fear of reinjury after recovery, thereby affecting sports performance and the risk of reinjury [72].

### 4.4. Types and Main Characteristics of Female Sports Injuries

#### 4.4.1. Gender Characteristics of ACL Injuries

This section provides a comprehensive overview of risk factors for ACL injuries in female athletes by integrating sex-based factors (such as hormonal, anatomical, and biomechanical characteristics) and gender-related factors (such as social and environmental influences). The ACL originates from the front of the intercondylar eminence of the tibia and attaches to the medial surface of the lateral femoral condyle. It is an important ligament that connects the femur and tibia and stabilizes the knee joint, and its main function is to limit excessive anterior translation and rotation of the tibia [73]. Therefore, athletes with ACL injuries will experience more difficulties in performing actions such as jumping, landing, and cutting when participating in sports that require high knee joint stability and multidirectional control, such as basketball and football. There are mainly three types of its injury: direct contact injury (accounting for about 30%), indirect contact injury, and noncontact injury (accounting for about 70%) [74].

ACL injury is one of the common sports injuries of athletes' knee joints [75]. The data of the National Collegiate Athletic Association shows that females, especially female gymnasts, basketball players, and football players, have a higher ACL injury rate [76]. For female athletes in different sports, the risk of ACL injury is 2 to 8 times that of male athletes [77–79]. The internal risk factors for female ACL injury include anatomical structure factors, such as smaller ACL size, width of the femoral intercondylar notch, larger posterior-inferior inclination of the lateral tibial plateau and body mass index, and larger knee joint laxity; neuromuscular and biomechanical factors, such as larger knee abduction and internal rotation angles, larger scoliosis amplitude, and more posteriorly shifted center of mass [11]; and hormonal factors. For example, in the preovulatory and follicular phases, due to the influence of estrogen on collagen metabolism, the laxity of the ligament increases and the stiffness decreases, resulting in a higher injury probability. However, this view has been challenged by the latest research [80].

#### 4.4.2. Gender Characteristics of BSIs

BSI is a type of sports injury caused by cumulative microdamage and overuse. It is mainly divided into three stages: bone strain or stress reaction, stress reaction, and stress fracture. In the first stage, medical imaging will show signs related to stress changes, but athletes usually have no pain symptoms; in the second stage, athletes will experience local bony pain, sometimes with tenderness on touch, and continued exercise will aggravate the symptoms; in the third stage, obvious fracture lines will appear in medical imaging, and athletes will feel obvious pain [81]. Regardless of gender, more severe injuries will lead to longer recovery times. The common sites of BSIs include the anterior or medial tibia, fibula, femoral neck, medial malleolus, and calcaneus [82].

Wentz et al. [25] pointed out in a systematic literature review that female athletes have a higher risk of stress fractures compared to male athletes. The average prevalence rates of stress fractures in female and male athletes are 9.7% and 6.5%, respectively. The risk factors for female BSIs include: older age, a history of previous BSIs [83], a history of amenorrhea or oligomenorrhea [84], and anatomical and biomechanical factors (smaller calf circumference, higher bony load-bearing rate, increased tibial moment, and increased femoral adduction angle) [85, 86] Orienteering competitions are also one of the causes of female BSIs [87]. Specifically, the wider pelvis of women leads to a larger anterior superior iliac spine patella center line and Q-angle, which in turn leads to an increased femoral adduction angle. In addition, the FAT of female athletes is also related to bone stress fractures [88].

#### 4.4.3. Gender Characteristics of Traumatic Brain Injuries

A concussion in sports, which is “a traumatic brain injury caused by biomechanical force,” and this biomechanical force may come from impacts on the face, neck or head, resulting in pathophysiological changes and affecting brain function. The clinical symptoms of concussion vary greatly, from obvious symptoms such as headache, dizziness, or loss of balance, to less common symptoms such as sleep disorders. It is worth noting that the relevant symptoms may occur at the moment of external force impact or may appear several minutes or hours after the impact [89].

According to the existing concussion epidemiology data, female athletes have a higher concussion IR compared to male athletes [26, 90–93]. Zuckerman et al. [26] found that among female athletes engaged in basketball, football, and softball, the concussion IRs were 53%, 83%, and 265% higher than those of male athletes, respectively. Although sports concussions often occur in impact or contact sports, women's lacrosse, as a noncontact sport, also has a considerably high concussion IR, which is 64% higher than that of men's lacrosse players. In addition, some studies have also pointed out that female athletes have more severe concussion symptoms, such as more severe cognitive function deficits and longer recovery times [94, 95]. The reason why women are more susceptible to concussions than men is not very clear, and the possible reasons can be explained from physiological and sociological perspectives. From a physiological perspective, the relationship between the neck circumference and related muscle strength of women and their head size and neck length is shorter and weaker than that of men, which may lead to a decrease in the stability and stiffness of the head and neck, thereby reducing the ability to absorb external stress [96, 97]. Brook et al. [90] and Tierney et al. [98] found that when dealing with functional external force loads, such as heading a ball, the head and neck of female athletes will have greater acceleration and displacement. From a sociological perspective, male athletes may conceal the occurrence of concussions due to concerns about not being able to participate in or continue the competition [99].

#### 4.4.4. Gender Characteristics of Nontraumatic Shoulder Joint Injuries

The glenohumeral joint is one of the joints with the greatest range of motion in the human body, and the balance between its stability and range of motion will affect the function of the shoulder joint. This balance is closely related to the combined effect of the restrictive function of the soft tissues around the shoulder joint, dynamic muscle strength, and bone anatomy. Shoulder joint instability (SJI) refers to the abnormal displacement of the humeral head in the glenoid cavity, accompanied by pain symptoms; according to the direction of the displacement of the humeral head, SJI can be divided into anterior, posterior, and multidirectional instability. The degree of instability also varies greatly, ranging from subluxation to complete dislocation [100]. According to the cause of occurrence, SJI can be divided into traumatic and nontraumatic types. The traumatic type is caused by external force, and about 69% of shoulder dislocations are due to this; the nontraumatic type is related to the internal structure of the shoulder joint and the laxity of the surrounding soft tissues. Some scholars have pointed out that a deeper and narrower glenoid cavity has a higher risk of joint instability [101], and the glenoid cavity morphology of women is smaller, with a larger inclination angle, and is generally more oval-shaped as a whole, while men present a more complete ball-and-socket joint [102], which makes women have a higher risk of nontraumatic SJI. Sports in which female athletes are more prone to SJI include rugby, basketball, swimming, overhead throwing events, and gymnastics [27, 103]. Hiemstra et al. (2002) [104] pointed out in a systematic literature review that among 2083 SJI patients, 22.3% were female; among 465 multidirectional SJI patients, 35.7% were female; among 247 posterior SJI patients, 27.1% were female. It is worth noting that general joint laxity is not necessarily related to shoulder joint laxity [105], and half of the female patients with multidirectional SJI have normal joint mobility in the Beighton scoring scale.

However, previous studies have found that college male athletes have a higher SJI IR than female athletes, with the former being 2.7 times that of the latter, and young men have the highest SJI risk [106, 107]. Goodman et al. [108] believed that this may be related to men's more frequent participation in sports with a higher impact risk, such as football and wrestling (Table 2).

## 5. Discussion

### 5.1. Main Findings Summary and Discussion

This scoping review elucidates the epidemiology of sports injuries among female athletes, highlighting key distinctions and challenges. Our findings indicate that while there is no significant overall difference in sports injury incidence between male and female athletes, specific injuries, including ACL injuries, BSIs, concussions, and nontraumatic shoulder instabilities, exhibit distinct gender-based epidemiological patterns and risk factors. Furthermore, this review identifies critical gaps in sports science and medicine research, such as the limited representation of female athletes in studies and the insufficient consideration of social gender factors.

The scarcity of female-specific research in sports science and medicine represents a significant barrier to optimizing athlete care. This “scarcity dilemma,” where female participants are vastly outnumbered by males in research, has profound clinical and practical consequences [5, 14]. For instance, the lack of data on how the menstrual cycle affects injury risk and recovery means that training and rehabilitation programs are often not tailored to the unique physiology of female athletes, potentially increasing their vulnerability to injury and prolonging recovery times [31]. This research gap extends to the understanding of conditions like the FAT and RED-S, where insufficient evidence may lead to underdiagnosis and mismanagement, with serious long-term health implications [6, 57–59]. The prevailing male-centric research model perpetuates a cycle where findings are inappropriately generalized to females, leading to suboptimal or even harmful prevention and treatment strategies.

**Figure 2:**
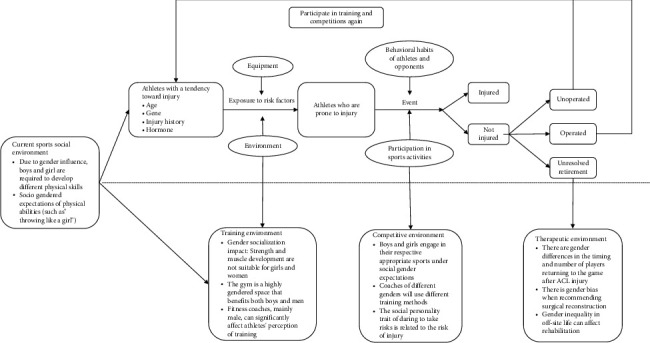
ACL injury cycle model combined with current social, training, competition, and treatment environments.

Furthermore, the underappreciation of social gender factors in injury etiology is a critical oversight. Our review highlights that gendered social norms and expectations can significantly influence an athlete's development, training environment, and health-seeking behaviors [18, 33, 34]. For example, societal pressures on body image can contribute to disordered eating and RED-S, while gender stereotypes in coaching may lead to inadequate strength and conditioning programs for girls and women, increasing their risk of injuries like ACL tears [17, 41, 42]. The proposed ACL Injury Cycle Model (Figure 2) provides a framework for understanding these complex interactions [11, 35]. It illustrates how the social environment, from early childhood development to the professional athletic sphere, shapes an athlete's risk profile. For healthcare professionals, this model underscores the need to look beyond purely biomechanical or physiological factors and to consider the broader social context in which injuries occur. For instance, in the therapeutic environment, gender bias can influence decisions about surgery and rehabilitation, affecting both physical and psychological recovery.

### 5.2. Female Sports Injuries and Clinical Nursing Practice

Firstly, it is necessary to clarify the research focus of female issues in sports science and sports medicine research, as well as the gap between the known and the unknown, and encourage the incorporation of gender factors into sports science and medical research. For example, in recent years, scholars from the United States, Canada, Australia, and the United Kingdom have jointly carried out a systematic review of research in the field of female SSSM. They described the review process in detail, including the gender composition of subjects, the athletic level of athletes, the statement of menstrual cycle, research topics, research impact factors, and sample size in previous studies, and proposed that research involving female subjects of “gold medal quality” should consider the number of days of the menstrual cycle, performance in different menstrual cycles, and the use of hormonal contraceptives [3]. Meanwhile, German scholars proposed that the relationship between female hormonal fluctuations and injury incidence should be considered, self-examination of potential biases should be carried out, and multidimensional and interdisciplinary research is also necessary [109]. It is worth mentioning that the journal Nature and the European Commission encourage the incorporation of gender-related analysis into research to establish a more inclusive research environment and make scientific research more comprehensive [110]. These suggestions are of great significance for promoting gender equality and conducting relevant research.

Secondly, a sports injury prevention and treatment model that explicitly integrates sex-based biological attributes with gender-related social factors should be established. For example, Meeuwisse et al. [35] proposed a multifactor, cyclical ACL injury treatment model. This model depicts that athletes with an injury-prone tendency, such as those with physiological-level sports injury risk factors like age, genes, injury history, and hormones, when exposed to an environment with a risk of sports injury such as equipment and venues, will develop into vulnerable athletes; during training or competition, affected by their own or opponents' behavior habits and sports participation situations, they may result in no injury or injury; after injury, they face different injury treatment decisions, such as whether to have surgery; those with poor recovery cannot continue to participate in sports, while athletes with good postinjury adaptation and recovery will return to the sports context and enter the above cycle again.

This model specifically introduces the influence of gender as a social construct across different contexts, including the presports, training, competitive, and therapeutic environments. For example, in the presports environment, it discusses whether boys and girls are required to develop different physical skills and whether different social gender expectations are given when developing physical abilities; in the training environment, it explores whether women are unable to actively train muscles and increase muscle strength due to the influence of social gender expectations, as well as the influence of the gym environment and the gender of fitness coaches; the competitive environment includes the influence of social gender on the sports events participated by men and women, the training methods of coaches of different genders, and the influence of different personality traits of men and women; the treatment environment includes the prognosis time point, injury treatment means, and off-field living conditions affected by social gender. This model emphasizes that the occurrence of sports injuries is the result of the interaction between athletes, the physical environment, and the social environment. This model constructs a thinking framework for dealing with sports injury problems, which not only provides clinical workers with an analysis idea of sports injuries other than biological sex but also provides researchers with a new research direction. That is, sports injury prevention research needs to adopt more complex methods, such as simultaneously including multiple elements such as social, environmental, and physiological, to explore the causes of sports injuries and promote their prevention and treatment.

In addition, for the traditional binary classification method of sports injury risk factors, a classification method of controllable and noncontrollable risk factors can be considered instead. For example, Parsons et al. [11] reorganized the risk factors of noncontact ACL injury according to the classification method of controllable and noncontrollable. The new classification method can remind clinical workers not to only focus on the individual factors of athletes but to analyze and solve injury protection problems from multiple angles according to the controllable attributes of risk factors and in combination with the influence of biological sex and social gender.

### 5.3. Implication for Nursing Management

The findings of this review have significant implications for nursing management in various healthcare settings that serve athletic populations. Nurse managers are in a key position to translate this evidence into practice to improve health outcomes for female athletes.

1. Education and Staff Development: Nurse managers should prioritize and facilitate ongoing education for nursing staff on the specific injury patterns and risk factors prevalent in female athletes, such as ACL injuries, BSIs, and concussions [25, 26, 76]. This training should include the influence of the menstrual cycle, hormonal fluctuations, and the signs and symptoms of RED-S on injury risk and recovery [22, 58, 59]. By ensuring their teams are well-informed, nurse managers can enhance the quality and specificity of care provided.
2. Development and Implementation of Clinical Protocols: Nurse managers can lead the development and implementation of evidence-based screening protocols to identify female athletes at high risk for certain injuries. These protocols could incorporate assessments of menstrual function [22, 57], nutritional status, biomechanical risk factors [11, 85, 86], and psychological well-being [24, 64, 65]. This proactive approach can facilitate early intervention and personalized injury prevention strategies.
3. Fostering Interdisciplinary Collaboration: Effective management of female athletes' health requires a collaborative, multidisciplinary approach. Nurse managers should work to break down silos and foster strong partnerships between nurses, physicians, physical therapists, dietitians, psychologists, and coaches. This ensures a holistic and coordinated approach to care that addresses the multifaceted nature of injury risk in female athletes.
4. Patient and Community Education: Nurse managers have a responsibility to ensure that nurses are empowered and equipped to provide effective education to female athletes, their families, and coaches. This education should cover topics such as injury prevention strategies, the importance of nutrition and energy availability, recognizing the signs of overtraining, and the destigmatization of mental health issues and help-seeking behaviors [69].
5. Advocacy and Research: Given the identified gaps in the literature, nurse managers should advocate for and support research that focuses on female athletes. This can be achieved by encouraging their staff to participate in data collection for institutional registries, supporting nursing-led research projects, and promoting a culture of inquiry. By championing research, nurse managers can contribute to building a more robust evidence base to guide the care of female athletes and challenge the historical underrepresentation of women in sports medicine research [5, 14].
6. Resource Allocation: Nurse managers should advocate for the allocation of resources toward the development and implementation of gender-specific injury prevention programs. This includes ensuring access to appropriate diagnostic tools and a network of specialized healthcare providers.

### 5.4. Limitations

This scoping review is subject to several limitations. The literature search was confined to electronic databases and high-impact sports science journals, which may have resulted in the omission of relevant studies published in less accessible sources. Some included studies examined broader athletic populations, including both male and female athletes across various sports, rather than focusing exclusively on female athletes, potentially reducing the specificity of the findings. Only English-language studies were included, which may have excluded relevant research published in other languages or from diverse cultural contexts. Additionally, variations in the definition and classification of sports injuries across different regions and studies may introduce inconsistencies. The review adopted a broad approach to sports injury epidemiology, incorporating both biological sex and social gender factors without adhering to a single theoretical framework, though researcher consensus was achieved. Moving forward, future studies should develop more precise frameworks for investigating gender-specific injury patterns, create targeted prevention and intervention strategies, and conduct rigorous randomized controlled trials to provide robust evidence for advancing injury prevention practices for female athletes.

## 6. Conclusions

This scoping review concludes that while overall sports injury incidence does not significantly differ, female athletes face distinct and higher-risk epidemiological patterns for key injuries, including ACL injuries, BSIs, and concussions. We assert that these disparities are compounded by critical gaps in sports science: namely, the underrepresentation of females in research and the pervasive neglect of social gender factors. To address these challenges, future research must integrate sex-based biological factors with gender-related sociocultural factors. Clinically, the findings call for targeted prevention strategies, wherein nursing management plays a pivotal role in leading education, implementing screening protocols, and fostering interdisciplinary care to better protect female athletes.

## Figures and Tables

**Table 1:** Basic information of included literature.

Author	Country	Type of study	Sample size	Research topic
Sallis et al. [8]	USA	Cohort study	3767	Comparative analysis of injury patterns between male and female athletes across seven collegiate sports.
Frisch et al. [1]	Luxembourg	Review	256	Gender-specific injury patterns and risk factors among young elite athletes.
Knox et al. [15]	USA	Cohort study	4.2 million	Surveillance of injury rates and analysis of injury patterns among US high school athletes.
Tone et al. [9]	Norwegian	Cohort study	1593	Six-year comparative study of injury risks and gender-specific injury patterns in World Cup alpine skiers.
Costello et al. [16].	Australia	Review	6,076,580	Evaluation of male-to-female participant ratios in sports and exercise medicine research
Cowley et al. [5].	Ireland	Review	12,511,386	Updated exploration of gender representation in sports and exercise science research.
Parsons and Ripat [17]	Canada	Review		Investigation of gender as a contributing factor in ACL injuries.
Fox et al. [18]	Australian	Review		Examination of environmental, sociocultural, and biological factors in ACL injuries.
Liederbach et al. [19]	USA	Prospective study	298	Epidemiology of ACL injuries among elite ballet and modern dancers.
Orishimo et al. [20]	USA	Cross-sectional study	40	Gender-based comparison of single-leg drop landing biomechanics between dancers and team sport athletes.
Vriend et al. [10]	USA	Systematic review	155 articles	Systematic classification of intervention strategies for acute sports injury prevention using the Haddon matrix to identify knowledge gaps.
Renstrom et al. [21]	Sweden	Review		Analysis of noncontact ACL injury factors in female athletes, including critical components of successful prevention programs and clinical management.
Cheng et al. [22]	USA	Cross-sectional study	1020	Association between menstrual dysfunction, hormonal contraceptive use, and bone stress injuries in US collegiate female athletes.
Nieves et al. [23]	USA	Cross-sectional study	72	Comparative study of bone dimensions and bone mass between genders with equivalent body size.
Walton et al. [24]	Australian	Cross-sectional study	523	Gender differences in mental health symptoms and risk factors among elite athletes.
Wentz et al. [25]	USA	Systematic review	21 articles	Systematic review of stress fracture incidence in military and athletic populations with gender-based etiological analysis.
Zuckerman et al. [26]	USA	Cohort study		Epidemiological characterization of concussions across 25 NCAA sports.
Patzkowski et al. [27]	USA	Cross-sectional study	36	Pathoanatomical assessment and gender-specific considerations in female shoulder instability cases.

**Table 2:** Classification of noncontact ACL injury factors.

	**Controllable factors**			**Uncontrollable factors**		

**External cause**	**Environment**	**Equipment**		**Environment**		

	Competition weather	Shoes		Competition situation		
	Competition venue	Kneepad		Opponent behavior		
	Competition rules			Random factors in the competition		
	Referee			Environment		
	Coach					

**Internal cause**	**Dissecting structure**	**Neuromuscular**	**Psychology**	**Dissecting structure**	**Hormone**	**Population statistics**

	Internal rotation of the foot	Dynamic knee valgus angle	Personality	Q-angle	Menstrual cycle	Age
	Body composition	Absolute muscle strength	Stress response	Structural knee valgus	Hormone concentration	Maturity level
	BMI	Relative muscle strength		Sinking of the navicular bone		History of ACL injury on the contralateral knee
		Muscle activation mode		Posture arrangement		Genetic factors
		Tendon strength		Size of femoral ankle incision		Gender
		Physical condition and muscle fatigue		ACL morphology and characteristics		Height
		Skill level		Tibial tilt angle		Race
		Neuromuscular control		Dissecting structure		Sport event
		Proprioceptive sensation				

## Data Availability

Data sharing is not applicable to this article as no datasets were generated or analyzed during the current study.

## Supporting Information

Supporting Information 1: Search Strategy.
