# Supplementary material for: Gender Differences in the Incidence and Types of Sports Injuries Among Female Athletes: A Scoping Review
Source: J Nurs Manag. 2025 Aug 30;2025:6416101. doi: 10.1155/jonm/6416101 (PMC12413956; doi:10.1155/jonm/6416101)
Supplement: Supporting Information — Additional supporting information can be found online in the Supporting Information section. [file 6416101.f1.docx]

Supplementary material 1 - Search Strategy

| Database | Search words |
| --- | --- |
| PubMed | (("Athletic Injuries"[Mesh] OR "sports injury*"[Title/Abstract] OR "athletic injury*"[Title/Abstract]) AND ("Sex Factors"[Mesh] OR gender[Title/Abstract] OR sex[Title/Abstract] OR male[Title/Abstract] OR female[Title/Abstract])) AND (incidence[Title/Abstract] OR prevalence[Title/Abstract] OR epidemiology[Title/Abstract] OR type*[Title/Abstract] OR specific*[Title/Abstract]) AND ("humans"[MeSH Terms]) |
| SportDiscus | (DE "Sports Injuries" OR TI "sports injury*" OR AB "sports injury*" OR TI "athletic injury*" OR AB "athletic injury*") AND (DE "Gender" OR DE "Sex Differences" OR TI gender OR AB gender OR TI sex OR AB sex OR TI male OR AB male OR TI female OR AB female) AND (TI incidence OR AB incidence OR TI prevalence OR AB prevalence OR TI epidemiology OR AB epidemiology OR TI type* OR AB type* OR TI specific* OR AB specific*) |
| Cochrane Library | ("sports injuries" OR "athletic injuries") AND (gender OR sex OR female OR male) AND (incidence OR prevalence OR epidemiology OR types) |
| Web of Science | TS=("sports injuries" OR "athletic injuries") AND TS=(gender OR sex OR male OR female) AND TS=(incidence OR prevalence OR epidemiology OR types) |
| Embase | ('sports injury'/exp OR 'sports injury*':ti,ab OR 'athletic injury*':ti,ab) AND ('sex difference'/exp OR gender:ti,ab OR sex:ti,ab OR male:ti,ab OR female:ti,ab) AND (incidence:ti,ab OR prevalence:ti,ab OR epidemiology:ti,ab OR type*:ti,ab OR specific*:ti,ab) |
| CINAHL | (MH "Athletic Injuries" OR TI "sports injury*" OR AB "sports injury*" OR TI "athletic injury*" OR AB "athletic injury*") AND (MH "Gender Issues" OR MH "Sex Factors" OR TI gender OR AB gender OR TI sex OR AB sex OR TI male OR AB male OR TI female OR AB female) AND (TI incidence OR AB incidence OR TI prevalence OR AB prevalence OR TI epidemiology OR AB epidemiology OR TI type* OR AB type* OR TI specific* OR AB specific*) |
